# Development and validation of a nomogram for predicting the outcome of metabolic syndrome among people living with HIV after antiretroviral therapy in China

**DOI:** 10.3389/fcimb.2025.1514823

**Published:** 2025-02-20

**Authors:** Yong Jin, Jiaona Zhu, Qingmei Chen, Mian Wang, Zhihong Shen, Yongquan Dong, Xiaoqing Li

**Affiliations:** Department of Infection, Ningbo Yinzhou No.2 Hospital, Ningbo, Zhejiang, China

**Keywords:** metabolic syndrome, nomogram, prediction model, HIV, aids

## Abstract

**Background:**

The prevalence of metabolic syndrome among people living with HIV (PLWH) is increasing worldwide. This study aimed to develop and validate a nomogram to predict the risk of metabolic syndrome in PLWH receiving antiretroviral therapy (ART) in China, accounting for both traditional and HIV-specific risk factors.

**Methods:**

A retrospective cohort study was conducted among PLWH receiving ART at a designated treatment center in Yinzhou District, China. A total of 774 patients were randomly assigned to development and validation cohorts in a 5:5 ratio. Predictive variables were identified using the least absolute shrinkage and selection operator and multivariable Cox regression analysis. The model’s discriminative ability was assessed using the C-index and the area under the receiver operating characteristic curve (AUC). Calibration was evaluated through calibration plots, and clinical utility was assessed using decision curve analysis (DCA).

**Results:**

﻿The nomogram incorporated age, ART regimen, body mass index, fasting blood glucose, high-density lipoprotein cholesterol, and HIV viral load as predictive factors. The C-index was 0.726 in the development cohort and 0.781 in the validation cohort, indicating strong discriminative ability. AUC values for predicting metabolic syndrome at 1, 2, and 3 years were 0.732, 0.728, and 0.737 in the development cohort, and 0.797, 0.803, and 0.783 in the validation cohort. Calibration plots showed strong concordance between predicted and observed outcomes, while DCA affirmed the model’s clinical applicability.

**Conclusion:**

A user-friendly nomogram incorporating six routinely collected variables was developed and internally validated, which can effectively predict metabolic syndrome in PLWH following ART.

## 1 Introduction

The introduction of standardized antiretroviral therapy (ART) has markedly lowered the incidence of opportunistic infections and mortality in people living with HIV (PLWH). However, metabolic disorders and related complications, including metabolic syndrome, cardiovascular disease (CVD), and diabetes, persist as major challenges. Metabolic syndrome, characterized by central obesity, impaired glucose tolerance, dyslipidemia, and hypertension, plays a key role in the incidence and progression of CVD in PLWH, ultimately contributing to increased mortality (Henning and Greene, 2023). A meta-analysis reported that the prevalence of metabolic syndrome in PLWH ranges from 16.7% to 31.3% across five continents (Nguyen et al., 2016), with particularly elevated rates observed in older adults (Sears et al., 2019). Early detection of metabolic syndrome continues to be a significant challenge.

Despite the known contributions of unhealthy behaviors, such as poor diet and reduced physical activity, PLWH face additional risk factors that increase their susceptibility to metabolic syndrome. While previous studies attributed metabolic syndrome primarily to ART drug effects, emerging evidence indicates that the viral infection itself may also contribute to its development (Zicari et al., 2019). A recent finding from Poland highlighted that metabolic syndrome prevalence is higher among PLWH than in the general population (Rogalska-Płońska et al., 2018). In addition, systematic reviews revealed that PLWH are at an increased risk of myocardial infarction, with risk factors including male sex, elevated HIV viral load, low CD4 count, high CD8 count, and specific ART regimens (Alsheikh and Alsheikh, 2022). In PLWH, the pathogenesis of metabolic syndrome may involve distinct mechanisms, including chronic immune activation, inflammation, and antiretroviral therapy, differing from those observed in the general population. Therefore, mitigating the incidence of metabolic syndrome in PLWH remains a major challenge.

The development of metabolic syndrome in PLWH is multifactorial and unpredictable by a single variable. Nomograms are widely used by clinicians to assess metabolic syndrome risk, and a systematic review has identified numerous clinically relevant predictors for the general population, including homeostasis model assessment insulin resistance index, serum insulin, free fatty acids, body weight, glycated albumin, hip circumference, mean corpuscular volume, mean corpuscular hemoglobin, physical activity, alanine aminotransferase (ALT), aspartate aminotransferase (AST), body mass index (BMI), neutrophilic granulocyte, total cholesterol (TC), uric acid (UA), low-density lipoprotein cholesterol (LDL-C), sex, smoking status, white blood cell count (WBC), lymphocyte, hemoglobin (HGB), hematocrit, and age (Zhang et al., 2020). However, the application of nomograms for predicting metabolic syndrome in PLWH following ART has been largely underexplored.

Given the significant differences in metabolic profiles between PLWH and the general population, concerns arise regarding the relevance of prediction models designed for healthy individuals. In our study, we aimed to identify several key factors influencing metabolic syndrome in PLWH and to develop a user-friendly nomogram to predict the likelihood of metabolic syndrome development following ART in this population.

## 2 Materials and methods

### 2.1 Data source and study population

**Figure 1:**
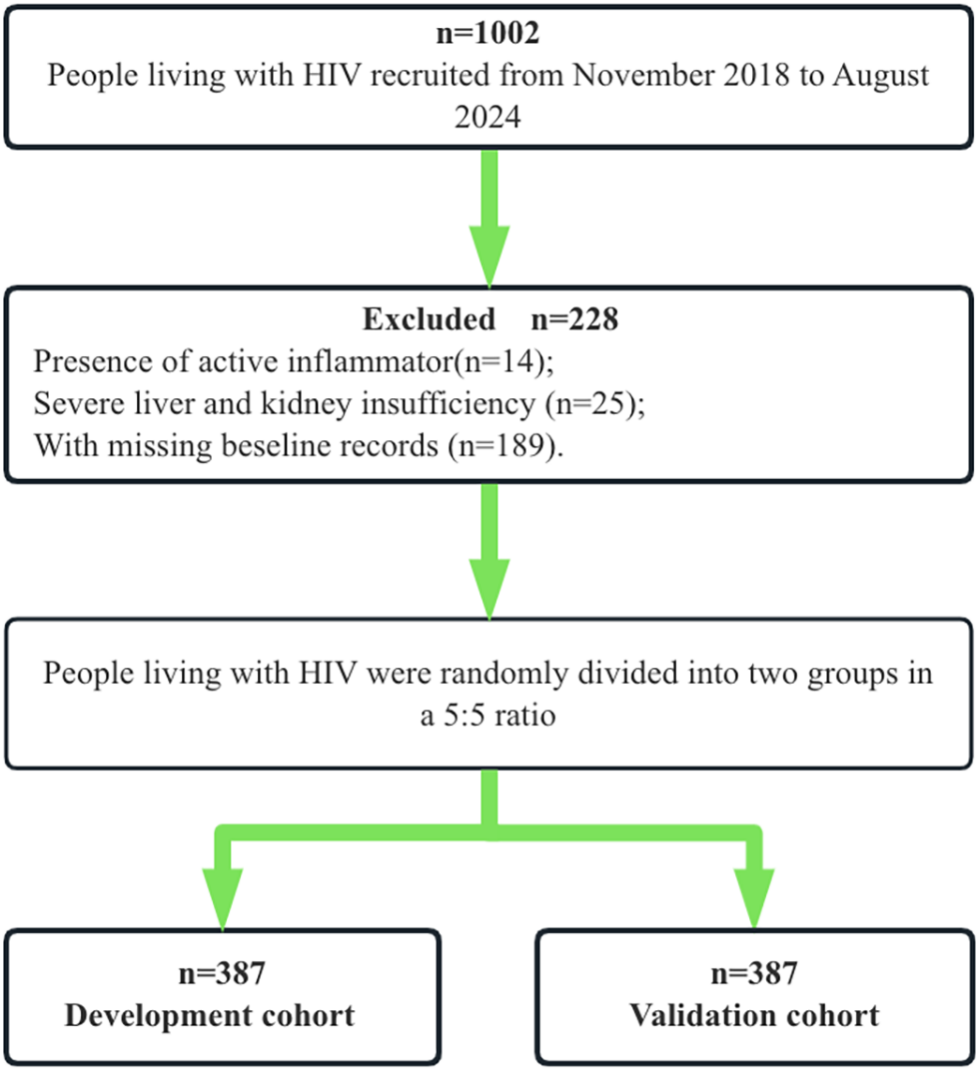
Flowchart of sample size selection.

The original database consisted of electronic medical records from 1002 PLWH treated at Ningbo Yinzhou No.2 Hospital between November 2018 and August 2024. Each patient initiated ART with a regimen consisting of two nucleoside reverse transcriptase inhibitors (NRTIs) and a third agent, which could be a non-nucleoside reverse transcriptase inhibitor (NNRTI), a protease inhibitor (PI), or an integrase strand transfer inhibitors (INSTI). Follow-up visits were scheduled at 2 weeks, 1, 2, and 3 months, and subsequently every 3 months thereafter. Based on this database, we performed a retrospective cohort study. Inclusion criteria were: (1) age ≥18 years at treatment initiation, (2) presence of HIV-1 infection, and (3) having at least one follow-up record that included terminal outcomes. Exclusion criteria were: (1) preexisting metabolic syndrome at baseline, (2) active inflammation as defined by the assessment of clinician at the initial visit, such as symptoms, signs, elevated inflammatory markers, and abnormal laboratory results, (3) neoplastic, or autoimmune diseases at the initial visit, (4) missing baseline records, or (5) pregnancy or planned pregnancy during the study period. A total of 774 participants who met the criteria were randomly assigned into a development cohort (n = 387) and a validation cohort (n = 387) in a 5:5 ratio (**Figure 1**).

The primary outcome of this study was the incidence of metabolic syndrome in PLWH. All participants resided in mainland China, and diagnostic criteria adhered to national guidelines, specifically those outlined in the “Guidelines for the Prevention and Treatment of Type 2 Diabetes” (Group et al., 2022; Fan et al., 2024). According to these criteria, metabolic syndrome is diagnosed when an individual presents three or more of the following five components: (1) Abdominal obesity, defined as a waist circumference (WC) ≥90 cm for men and ≥ 85 cm for women; (2) Hyperglycemia, defined as fasting blood glucose (FBG) ≥ 6.1 mmol/L, 2-hour post-glucose load blood glucose ≥ 7.8 mmol/L, or a diagnosis of diabetes; (3) Hypertension, defined as blood pressure ≥ 130/85 mmHg or previously diagnosed and treated hypertension; (4) Elevated fasting triglycerides (TG) ≥1.70 mmol/L; and (5) Low fasting high-density lipoprotein cholesterol (HDL-C) <1.04 mmol/L.

### 2.2 Study variables

The baseline variables were defined as the first examination conducted at our hospital, and variables associated with the metabolic syndrome incidence were selected as candidate variables. Detailed medical history, including information on drug use, disease history, age, gender, and transmission category, was provided by each participant. There are three transmission categories in this study: men who have sex with men (MSM), heterosexuals, and those who prefer not to say. Complete physical examination data included height, weight, WC, systolic blood pressure (SBP), and diastolic blood pressure (DBP). BMI was calculated as body weight (kg) divided by height squared (m²). Following at least eight hours of fasting, general laboratory parameters were assessed including FBG, ALT, AST, indirect bilirubin (IBIL), direct bilirubin (DBIL), albumin (ALB), globulin (GLB), UA, creatinine (CREA), blood urea nitrogen (BUN), TC, TG, HDL-C, LDL-C, HGB, platelets (PLT), WBC, CD4 T cell count, CD8 T cell count, CD4/CD8 ratio, and HIV viral load.

### 2.3 Ethics approval and consent to participate

This study was approved by the Institutional Review Board of Ningbo Yinzhou No.2 Hospital (Approval No.: 2023-050). Each patient provided informed consent at ART initiation, permitting the use of clinical records for future epidemiological studies. No additional consent was required, and all clinical records were deidentified prior to analysis. We signed a confidentiality agreement and were authorized to access the database for this study.

### 2.4 Statistical analysis

Statistical analyses were performed using R software (v4.3.2) and IBM SPSS Statistics (v24.0). Prior to statistical analysis, all parameters were tested for normality using the Kolmogorov-Smirnov test. All continuous variables were non-normally distributed and are expressed as the median (interquartile range, IQR). Non-normally distributed variables between the two groups were compared using the Mann-Whitney U test. Categorical variables were expressed as counts and percentages, with group comparisons conducted using the Chi-square test or Fisher’s exact test, as appropriate. Statistical significance was defined as a two-tailed P-value <0.05.

### 2.5 Predictor selection

The least absolute shrinkage and selection operator (LASSO) method, applied via the “glmnet” package, was used to identify the key predictive variables from the primary dataset (Sauerbrei et al., 2007). LASSO is a regularization technique for variable selection and shrinkage, ensuring that only the most relevant features are retained.

### 2.6 Construction of the nomogram

The prognostic model was developed in the development cohort using a stepwise approach. Initially, univariate Cox regression analyses were conducted to identify significant variables (age, ART regimen, SBP, WC, BMI, FBG, ALB, GLB, UA, HDL-C, and HIV viral load) with P-values <0.05, which were then included in multivariate analyses. The final nomogram was constructed based on the results of stepwise multivariate Cox regression, visually representing the model by assigning risk scores to each variable axis and calculating incidence of metabolic syndrome probabilities for 1, 2, and 3 years using weighted regression coefficients and the baseline survival function. A scoring system was developed from standardized regression coefficients to score each variable. The nomogram was plotted using the “rms” and “survival” packages. To assess predictive accuracy, Harrell’s C-index was calculated, and bootstrapping validation with 1,000 resamples was performed to obtain a corrected C-index.

### 2.7 Validation of model performance

The nomogram’s discrimination and calibration ability were evaluated using the receiver operating characteristic (ROC) curve and calibration plot in both the development and validation cohorts. The “timeROC” package was used to calculate the area under the curve (AUC) based on sensitivity and specificity values. Calibration was assessed using a plot with a 45° diagonal line, generated from 1,000 bootstrap resamples, indicating the agreement between predicted and actual incidence of metabolic syndrome at specific time points. To evaluate the model’s clinical utility, decision curve analysis (DCA) was performed using the “rmda” package. This method plots net benefit over a range of threshold probabilities to demonstrate the model’s potential for clinical decision-making (Vickers et al., 2019).

## 3 Results

### 3.1 Participant characteristics

In this study, participants were randomly assigned to the development and validation cohorts in a 5:5 ratio with each consisting of 387 PLWH. The demographic characteristics were comparable between the two cohorts (**Table 1**). After a median follow-up of 1.9 (0.4, 3.5) years, the overall incidence of metabolic syndrome was found to be 27.3%. During follow-up, 107 patients in the development cohort and 104 in the validation cohort developed metabolic syndrome.

**Table 1:** Demographic characteristics of people living with HIV in development and validation cohorts.

﻿Variable	﻿﻿Development cohort (n=387)	﻿Validation cohort (n=387)	﻿P-value
Gender (n), %			0.453
Male	333 (86.0)	341 (88.1)	
Female	54 (14.0)	46 (11.9)	
Age (years), median (IQR)	36 (28,46)	36 (28,47)	0.889
Transmission category (n), %			0.465
Unknown	33 (8.5)	30 (7.8)	
MSM	182 (47.0)	199 (51.4)	
Heterosexual	172 (44.4)	158 (40.8)	
ART regimen (n), %			0.622
NNRTIs-based	262 (67.7)	249 (64.3)	
PIs -based	15 (3.9)	17 (4.4)	
INSTIs-based	110 (28.4)	121 (31.3)	
SBP (mmHg), median (IQR)	121.2 (118.8,123.3)	121.1 (119.1,123.8)	0.528
DBP(mmHg), median (IQR)	76.2 (72.8,79.8)	75.9 (72.1,80.1)	0.838
WC (cm), median (IQR)	79.1 (76.7,81.0)	78.8 (76.5,81.2)	0.787
BMI (﻿kg/m^2^), median (IQR)	21.9 (21.4,22.3)	21.9 (21.3,22.2)	0.773
FBG (mmol/L), median (IQR)	5.4 (5.2,5.6)	5.4 (5.2,5.6)	0.537
ALT (﻿U/L), median (IQR)	32.6 (21.0,34.6)	32.6 (21.0,35.2)	0.703
AST (U/L), median (IQR)	25.0 (20.0,28.0)	24.8 (20.0,27.1)	0.392
IBIL (μmol/L), median (IQR)	6.1 (4.3,6.5)	6.1 (4.5,6.5)	0.509
DBIL (μmol/L), median (IQR)	2.1 (1.6,2.5)	2.1 (1.6,2.5)	0.357
ALB (g/L), median (IQR)	46.2 (44.6,47.8)	46.5 (44.6,47.9)	0.483
GLB (g/L), median (IQR)	30.3 (28.1,33.0)	30.7 (27.8,34.0)	0.484
UA (μmol/L), median (IQR)	349.8 (315.4,379.5)	347.7 (308.9,381.1)	0.986
CREA (μmol/L), median (IQR)	67.4 (61.5,73.2)	67.8 (60.0,73.2)	0.462
BUN (mmol/L), median (IQR)	4.6 (4.2,5.0)	4.6 (4.0,5.1)	0.738
TC (mmol/L), median (IQR)	4.4 (4.2,4.7)	4.4 (4.2,4.7)	0.693
TG (mmol/L), median (IQR)	1.7 (1.3,1.8)	1.7 (1.3,1.9)	0.480
HDL-C (mmol/L), median (IQR)	1.1 (1.1,1.2)	1.1 (1.1,1.2)	0.831
LDL-C (mmol/L), median (IQR)	2.6 (2.4,2.8)	2.6 (2.4,2.8)	0.445
HGB (g/L), median (IQR)	149.0 (143.0,155.0)	149.1 (143.7,155.0)	0.859
PLT (10^9^/L), median (IQR)	221.3 (199.0,236.0)	221.9 (202.3,242.0)	0.314
WBC (10^9^/L), median (IQR)	5.2 (4.6,5.6)	5.2 (4.6,5.7)	0.817
CD4 T cell count (﻿cells/μL), median (IQR)	427.0 (361.3,485.0)	423.8 (364.8,491.8)	0.612
CD8 T cell count (cells/μL), median (IQR)	683.0 (586.0, 740.2)	691.9 (655.6,716.7)	0.253
CD4/CD8 ratio, median (IQR)	0.64 (0.51,0.78)	0.62 (0.53,0.73)	0.440
HIV viral load (log copies/mL), median (IQR)	1.0 (1.0,3.0)	1.0 (1.0,2.7)	0.275

MSM, men who have sex with men; ART, antiretroviral therapy; NNRTIs, non-nucleoside reverse transcriptase inhibitors; PIs, protease inhibitors; INSTIs, integrase strand inhibitors; SBP, systolic blood pressure; DBP, diastolic blood pressure; WC, waist circumference; BMI, body mass index; FBG, fasting blood glucose; ALT, alanine aminotransferase; AST, aspartate aminotransferase; IBIL, indirect bilirubin; DBIL, direct bilirubin; ALB, albumin; GLB, globulin; UA, uric acid; CREA, creatinine; BUN, blood urea nitrogen; TC, total cholesterol; TG, triglyceride; HDL-C, high-density lipoprotein cholesterol; LDL-C, low-density lipoprotein cholesterol; HGB, hemoglobin; PLT, platelets; WBC, white blood cell.

### 3.2 Selection of prognostic predictors

In the development cohort, 29 variables, including gender, age, transmission category, ART regimen, SBP, DBP, WC, BMI, FBG, ALT, AST, IBIL, DBIL, ALB, GLB, UA, CREA, BUN, TC, TG, HDL-C, LDL-C, HGB, PLT, WBC, CD4 T cell count, CD8 T cell count, CD4/CD8 ratio, and HIV viral load, were included in the LASSO regression analysis.

**Figure 2:**
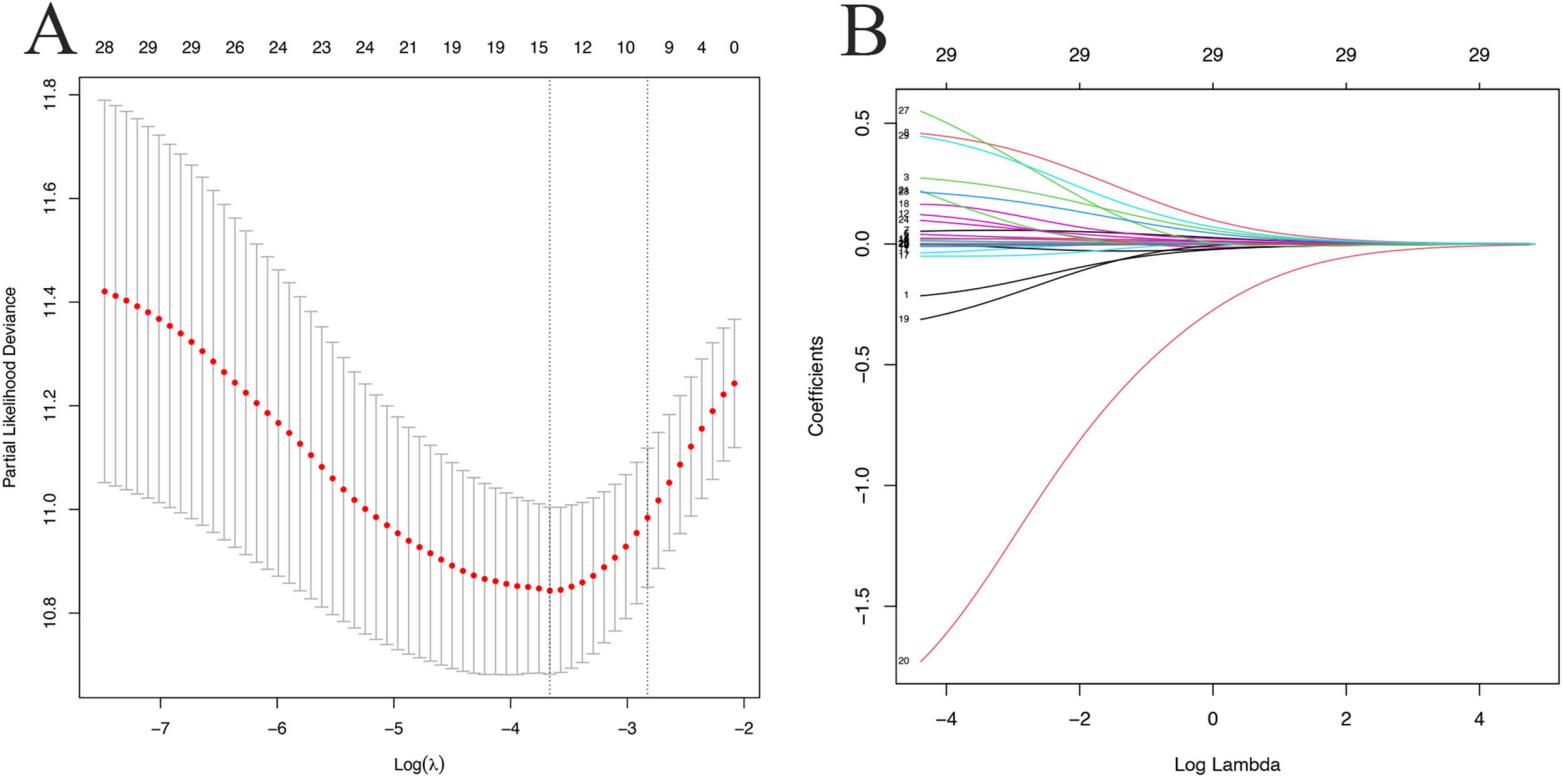
LASSO logistic regression plots. **(A)** Partial likelihood deviance plot; **(B)** LASSO coefficient profiles. Each colored curve represents the LASSO coefficient profile of a feature plotted against the Log (λ) sequence. The numbers above the graph indicate the number of variables included in the model at each corresponding value of λ on the x-axis.

The “glmnet” package in R software was employed with the “family = cox” parameter. As shown in **Figure 2**, as λ increased, the variable coefficients decreased progressively. Subsequently, the optimal λ parameter was identified through cross-validation with Lambda.1se set at 0.0592, as shown in **Figure 2**. After applying the optimal parameter values to the model, variables with nonzero coefficients were identified in the LASSO logistic regression model (**Supplementary Table S1**). Ultimately, a total of 11 independent variables were identified as optimal predictors, including age, ART regimen, SBP, WC, BMI, FBG, ALB, GLB, UA, HDL-C and HIV viral load.

### 3.3 Prognostic model construction

Based on the findings, variables identified by LASSO regression analysis and deemed clinically relevant (including TC, TG, and LDL-C) were initially included in a univariate Cox regression model. Variables except TC, TG, and LDL-C were significantly associated with the development of metabolic syndrome (P < 0.05). In the stepwise multivariate analysis that followed, SBP, WC, ALB, GLB, UA, TC, TG, and LDL-C were removed to optimize the model(P>0.05). The final model, consisting of six variables (age, ART regimen, BMI, FBG, HDL-C, and HIV viral load), was developed to predict the occurrence of metabolic syndrome in PLWH (**Table 2**).

**Table 2:** Univariable and multivariable Cox analysis of metabolic syndrome in the development cohort.

Variable	﻿Univariable Cox regression model		﻿Multivariate Cox regression model	
	HR (95% CI)	P-value	﻿ HR (95% CI)	P-value
Age	1.032 (1.018-1.045)	<0.001	1.028 (1.014-1.042)	<0.001
ART regimen				
NNRTIs-based	Ref.		Ref.	
PIs-based	0.447 (0.109-1.829)	0.263	0.431 (0.103-1.804)	0.249
INSTIs-based	2.432 (1.593-3.713)	<0.001	3.021 (1.962-4.653)	<0.001
SBP	1.092 (1.060-1.124)	<0.001		
WC	1.073 (1.038-1.109)	<0.001		
BMI	1.174 (1.081-1.276)	<0.001	1.119 (1.018-1.230)	0.020
FBG	1.569 (1.216-2.023)	0.001	1.437 (1.069-1.930)	0.016
ALB	0.895 (0.855-0.937)	<0.001		
GLB	1.074 (1.041-1.108)	<0.001		
UA	1.003 (1.001-1.005)	0.001		
TC	1.211 (0.922-1.590)	0.169		
TG	1.075 (0.927-1.247)	0.341		
HDL-C	0.159 (0.055-0.460)	0.001	0.225 (0.078-0.652)	0.006
LDL-C	1.076 (0.743-1.558)	0.698		
HIV viral load	1.273 (1.126-1.441)	<0.001	1.257 (1.108-1.425)	<0.001

ART, antiretroviral therapy; NNRTIs, non-nucleoside reverse transcriptase inhibitors; PIs, protease inhibitors; INSTIs, integrase strand inhibitors; SBP, systolic blood pressure; WC, waist circumference; BMI, body mass index; FBG, fasting blood glucose; ALB, albumin; GLB, globulin; UA, uric acid; TC, total cholesterol; TG, triglyceride; HDL-C, high-density lipoprotein cholesterol; LDL-C, low-density lipoprotein cholesterol.

**Figure 3:**
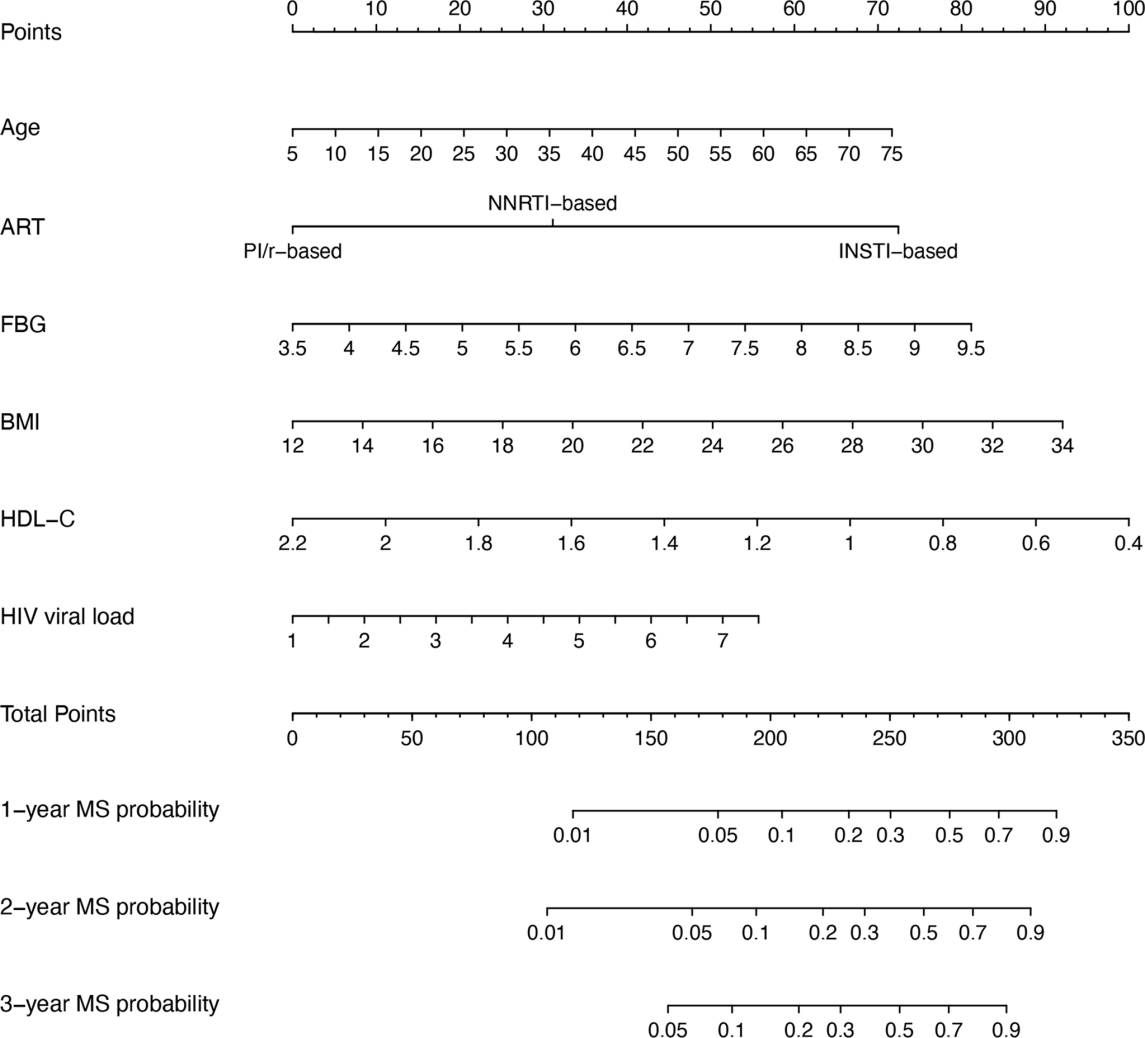
Nomogram for predicting metabolic syndrome in PLWH. To use the nomogram, a line is drawn from each parameter value to the corresponding score on the score axis. Sum the points for all parameters, then draw a line from the total score to determine the likelihood of metabolic syndrome, shown on the bottom line of the nomogram. ART, antiretroviral therapy; BMI, body mass index; FBG, fasting blood glucose; HDL-C, high-density lipoprotein cholesterol; MS, metabolic syndrome.

**Figure 3** outlines the nomogram usage protocol. The process is as follows: (1) Construct the nomogram: Regression coefficient of each variable from the stepwise multivariate Cox regression analysis was proportionally transformed to a scale ranging from 0 to 100. The variable with the highest absolute β coefficient was designated as 100%. (2) Locate the position of each variable on the corresponding axis and draw a line straight upward to the Points axis to determine the score associated with that variable. (3) Add the points from all variables to obtain the total score. (4) Draw a line from the total points axis straight down to the corresponding 1-, 2-, or 3-year metabolic syndrome probability axis to determine the predicted probability of metabolic syndrome. A higher total score indicated an increased risk of developing metabolic syndrome in PLWH after ART.

### 3.4 Discrimination and calibration of the prognostic model

**Figure 4:**
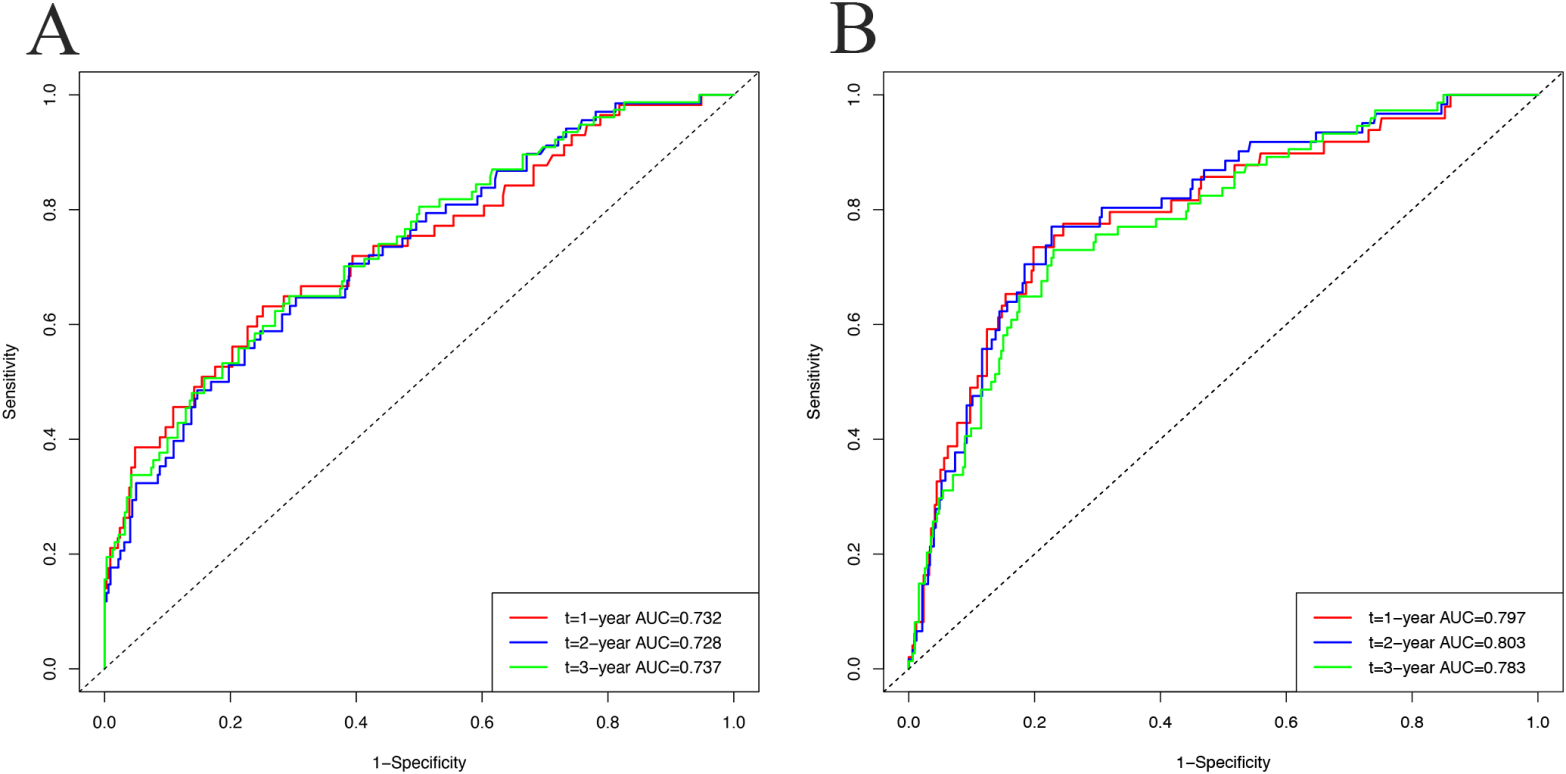
The receiver operating characteristic (ROC) curves and the area under the ROC curves (AUC) to predict metabolic syndrome incidence at 1, 2, and 3 years in the development cohort **(A)** and validation cohort **(B)**.

In the development cohort, the C-index was 0.726 (95% CI, 0.680-0.790), and the AUC values for 1, 2, and 3 years were 0.732 (95% CI, 0.656-0.808), 0.728 (95% CI, 0.660-0.795), and 0.737 (95% CI, 0.673-0.801), respectively. For the validation cohort, the C-index was 0.781 (95% CI, 0.726-0.831), and the AUC values for 1, 2, and 3 years were 0.797 (95% CI, 0.727-0.867), 0.803 (95% CI, 0.743-0.863), and 0.783 (95% CI, 0.725-0.841), respectively. **Figures 4A, B** shows the ROC curves for these analyses.

**Figure 5:**
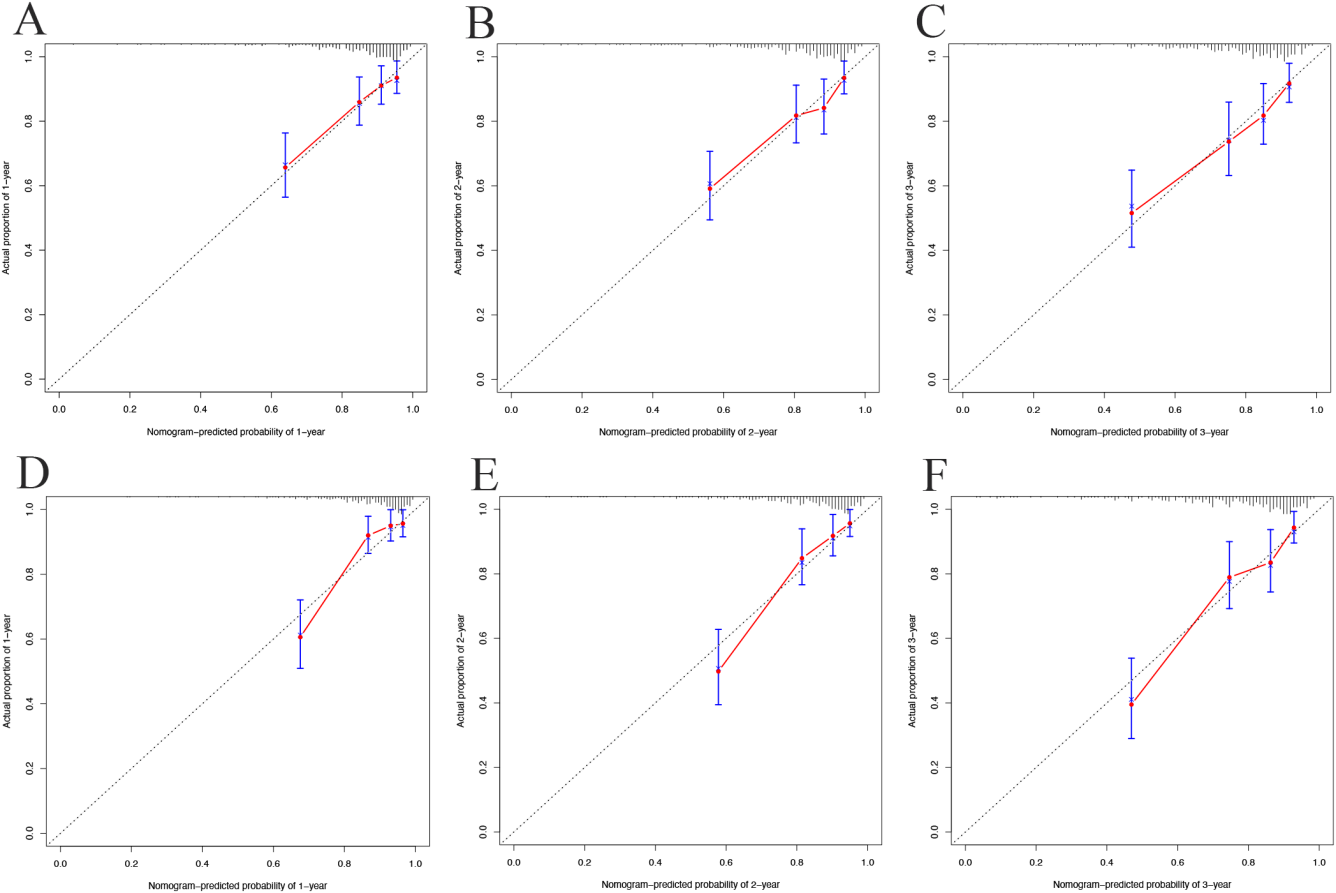
Calibration curves for 1-year **(A)**, 2-year **(B)**, and 3-year **(C)** in the development cohort; 1-year **(D)**, 2-year **(E)**, and 3-year **(F)** in the validation cohort.

Calibration plots showed strong concordance between predicted and actual cases of metabolic syndrome in both cohorts. In the development cohort (**Figures 5A–C**) and validation cohort (**Figures 5D–F**), the curves closely aligned with the 45° line, indicating good model calibration.

### 3.5 Decision curve analysis of the prognostic model

**Figure 6:**
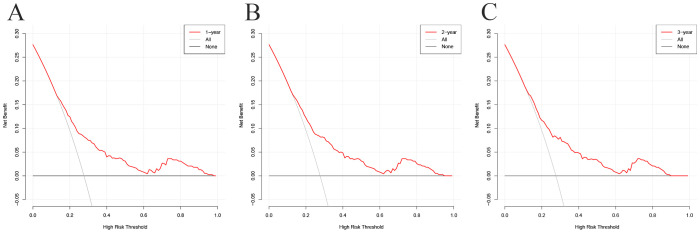
Decision curve analysis (DCA) curves for the prognostic model at 1-year **(A)**, 2-year **(B)**, and 3-year **(C)** in the development cohort.

In the decision curve analysis (DCA), the x-axis represents the threshold probability, and the y-axis represents the net benefit. As illustrated in **Figure 6**, the threshold probability in the development cohort ranged from 0% to 97% at 1 year, 0% to 94% at 2 years, and 0% to 89% at 3 years. The wide threshold range across all three time points indicates that the model provides higher net benefits across nearly all reasonable threshold probabilities, indicating its potential utility in diverse clinical decision-making scenarios.

## 4 Discussion

In this retrospective study, routine clinical data related to metabolic syndrome in PLWH were identified using LASSO and multivariable Cox regression analyses. Our model incorporates HIV-specific variables, such as ART regimen and viral load, making it a significant advancement compared to previous prediction models. Ultimately, age, ART regimen, BMI, FBG, HDL-C, and HIV viral load were selected to construct a predictive nomogram for metabolic syndrome outcomes in PLWH after ART. The ROC curves, calibration plots, and DCA demonstrated excellent discrimination and calibration of the model, while internal validation confirmed its strong applicability and generalizability. Early risk assessment of metabolic syndrome in PLWH enables timely and individualized intervention.

Our findings reveal that metabolic syndrome is prevalent in the Chinese PLWH cohort, with an overall incidence of 27.3% after an average follow-up of 1.9 (0.4, 3.5) years. Notably, the prevalence exceeds that reported in the general population, as indicated in previous literature (Bonfanti et al., 2007; Rogalska-Płońska et al., 2018). Certain variables, such as age, BMI, FBG, and HDL-C, have been shown to predict metabolic syndrome in non-HIV-infected individuals. And the prevalence of metabolic syndrome increases with age across various populations (Nix and Tien, 2014). Weight gain is particularly significant in PLWH, often indicating an elevated risk of diabetes and CVD (Achhra et al., 2016; Herrin et al., 2016). Reduced HDL levels, a surrogate marker for metabolic syndrome, have been validated in PLWH as reliable predictors of cardiovascular disease and other metabolic abnormalities (Sun et al., 2024). Although variables like SBP, ALB, and TC have shown predictive value for metabolic syndrome in non-HIV-infected individuals (Zhang et al., 2020), they did not demonstrate such predictive value in PLWH in our study. This suggests that although some risk factors are shared, the underlying mechanisms of metabolic syndrome in PLWH may differ from those in the general population. Alongside traditional risk factors, HIV-specific factors also significantly contribute to the development of metabolic syndrome. It has been shown that immune activation and chronic inflammation associated with HIV are key contributors (Hsu and Sereti, 2016), and the side effects of ART, particularly from INSTI-based (Jemal, 2024) and PIs-based (Echecopar-Sabogal et al., 2018) therapies, have been associated with metabolic complications.

Our predictive model highlights baseline viral load as a key predictor. Viral load is a crucial marker for managing the health of PLWH, correlating with not only opportunistic infection (Swindells et al., 2002), but also metabolic disorders like metabolic syndrome (Jacobson et al., 2006; Squillace et al., 2009; Hamooya et al., 2021; Han et al., 2022), hypertension (Martin-Iguacel et al., 2016), glucose dysregulation (El-Sadr et al., 2005), and hyperlipidemia (Sun et al., 2020). A high baseline viral load suggests a prolonged time to achieve viral suppression. Chronic HIV infection, particularly when viral load is poorly controlled, leads to systemic inflammation and immune activation, which are potential drivers of metabolic disturbances (Hsu and Sereti, 2016). The virus itself can disrupt lipid metabolism by affecting pathways involving adipocytes and liver function, resulting in abnormal lipid profiles frequently seen in these patients (Agarwal et al., 2013). Research indicates that individuals with uncontrolled HIV viral loads face a significantly higher risk of developing metabolic syndrome than those with suppressed viral loads, even after adjusting for other risk factors such as age, ART regimen, and lifestyle (Bonfanti et al., 2010). This highlights that viral load control is vital not only for preventing immune decline but also for reducing the risk of metabolic syndrome and related complications, such as CVD. Therefore, regular viral load monitoring is essential in managing PLWH to lower the risk of metabolic syndrome.

INSTIs have become increasingly linked to metabolic syndrome in PLWH, with those on INSTI-based treatment experiencing significant weight gain, particularly visceral fat accumulation—a hallmark of metabolic syndrome (Eckard and McComsey, 2020; Ando et al., 2021; Jemal, 2024). This weight gain tends to occur rapidly after therapy initiation and is often accompanied by heart failure, hypertension, myocardial infarction, lipid disorders, and other metabolic conditions (Byonanebye et al., 2022; Rebeiro et al., 2023). INSTIs are believed to alter adipocyte differentiation and disrupt energy metabolism, leading to excessive fat deposition. In animal studies, INSTI treatment in non-infected macaques resulted in increased fibrosis, adipocyte enlargement, and enhanced adipogenesis in both subcutaneous and visceral adipose tissue (Gorwood et al., 2020). *In vitro*, adipocyte hypertrophy induced by INSTI exposure contributes to fat fibrosis through hypoxia, initiating a cascade that further amplifies hypertrophy and is strongly associated with insulin resistance development (Ngono Ayissi et al., 2022). INSTIs also lowered adiponectin and leptin production, increased interleukin-6 levels, and mildly suppressed adipogenesis markers, indicating a detrimental effect on adipose tissue metabolism and inflammation (Domingo et al., 2022). Although both viral load and INSTIs contributed to metabolic syndrome, their components differ. INSTIs primarily contributed to metabolic syndrome by promoting weight gain (Eckard and McComsey, 2020; Ando et al., 2021; Jemal, 2024), whereas HIV infection itself was more closely associated with lipid abnormalities (Agarwal et al., 2013). Thus, differences in the predominant features of metabolic syndrome might provide indirect evidence to distinguish between the contributions of HIV and ART drugs. Meanwhile, the contributions of various factors differed. As shown in **Figure 3**, the scores assigned to INSTIs exceeded those for viral load, suggesting that ART drugs might have a greater influence than viral load. As such, patients on INSTI-based regimens should be closely monitored for metabolic syndrome, particularly those with pre-existing risk factors like obesity, hypertension, or dyslipidemia. Although INSTI-based drugs like Biktarvy can rapidly suppress the virus, this benefit does not seem to outweigh their adverse metabolic effects in our study. Interestingly, while PIs have historically been associated with metabolic abnormalities, they do not appear to increase the risk of metabolic syndrome compared to NNSTIs in our study. PIs are known to contribute to dyslipidemia, particularly elevated triglyceride and cholesterol levels (Lagathu et al., 2019), as well as insulin resistance (Kajogoo et al., 2021). However, it is important to note that while PIs undeniably cause lipid metabolism abnormalities, they do not necessarily lead to full-blown metabolic syndrome. The diagnosis of metabolic syndrome requires multiple criteria beyond dyslipidemia, such as increased waist circumference, hypertension, and elevated fasting glucose levels. This has also been observed in cross-sectional studies, where INSTIs, rather than PIs, were the main contributors to metabolic syndrome (Hamooya et al., 2021). Despite this, PIs remain a critical part of ART in certain contexts, but their long-term metabolic risks necessitate regular monitoring of lipid and glucose levels. As with INSTIs, balancing effective viral suppression with the management of metabolic side effects is key to optimizing patient outcomes. In cases of high-risk metabolic syndrome, switching to a more metabolic-friendly ART regimen may be necessary.

The superiority of LASSO regression analysis over traditional methods that rely on univariate correlation strength for predictor selection is well-recognized (Jiang et al., 2016). In our study, the Lambda.1se output identified a high-performing model with fewer independent variables, providing an optimal approach for selecting current risk factors. Nomograms are valuable tools for visualizing predictive models. Based on LASSO and multivariable Cox regression results, we developed a nomogram incorporating six key variables: age, ART regimen, BMI, FBG, HDL-C, and HIV viral load. The C-index and AUC demonstrate that this nomogram has excellent discriminatory ability in both the development and validation cohorts. Moreover, calibration plots and DCA confirm the model’s high accuracy and clinical utility based on the development cohort. This study has several notable strengths, one of which is that it pilots to identify HIV-specific factors that existing models often neglect. By incorporating these critical variables, our nomogram offers a more accurate and tailored tool for assessing metabolic syndrome risk in PLWH. Additionally, all predictive factors are readily measured in routine clinical practice, enhancing the model’s practicality for real-world application. This ensures healthcare providers can readily use the nomogram in real-world settings to improve risk assessment and management of metabolic syndrome in PLWH, ultimately enhancing patient outcomes.

Our institution serves as the designated HIV treatment center in Yinzhou District, and the study population closely represents the local PLWH. However, there are several limitations to our study. First, this is a single-center study without external validation, necessitating caution when generalizing the findings to other populations. Second, the limited sample size prevented a detailed analysis of specific antiretroviral drugs, particularly PIs. Furthermore, metabolic syndrome is a multifactorial condition, and our model did not account for factors such as family history of comorbidities, diet, physical activity, smoking history, or alcohol consumption, limiting its robustness. These limitations prevent us from making more robust adjustments to the prediction model. Future large-scale, prospective studies are necessary to validate our findings and to refine the predictive model for metabolic syndrome in PLWH.

## 5 Conclusion

Our study identifies age, ART regimen, BMI, FBG, HDL-C, and HIV viral load as significant risk factors for the development of metabolic syndrome in PLWH after ART. Based on these findings, a straightforward and user-friendly nomogram that integrates these six variables to predict the risk of metabolic syndrome in this population was constructed and validated. While this model shares some risk factors with the established one targeting the general population, there are also critical differences. Comprehensive management of metabolic syndrome in PLWH should consider both the ART regimen and viral load.

## Data Availability

The raw data supporting the conclusions of this article will be made available by the authors, without undue reservation.
