# Supplementary material for: Development and validation of a nomogram for predicting the outcome of metabolic syndrome among people living with HIV after antiretroviral therapy in China
Source: Front Cell Infect Microbiol. 2025 Feb 20;15:1514823. doi: 10.3389/fcimb.2025.1514823 (PMC11882517; doi:10.3389/fcimb.2025.1514823)
Supplement: Supplementary file 1 [file Table1.docx]

Table S1 𝜆 of LASSO regression analysis

| No. | Variable | 𝜆 |
| --- | --- | --- |
| 1 | Gender | 0 |
| 2 | Age | 0.0106837068 |
| 3 | Transmission category | 0 |
| 4 | SBP | 0.026303893 |
| 5 | DBP | 0 |
| 6 | WC | 0.0012989136 |
| 7 | BMI | 0.0408895728 |
| 8 | FBG | 0.0911762235 |
| 9 | ALT | 0 |
| 10 | AST | 0 |
| 11 | IBIL | 0 |
| 12 | DBIL | 0 |
| 13 | ALB | -0.0255021617 |
| 14 | GLB | 0.0104682359 |
| 15 | UA | 0.0002632649 |
| 16 | CREA | 0 |
| 17 | BUN | 0 |
| 18 | TC | 0 |
| 19 | TG | 0 |
| 20 | HDL-C | -0.0512602573 |
| 21 | LDL-C | 0 |
| 22 | HGB | 0 |
| 23 | PLT | 0 |
| 24 | WBC | 0 |
| 25 | CD4 T cell count | 0 |
| 26 | CD8 T cell count | 0 |
| 27 | CD4/CD8 | 0 |
| 28 | HIV viral load | 0.0628279274 |
| 29 | ART regimen | 0.1008153668 |

Abbreviations: SBP, systolic blood pressure; DBP, diastolic blood pressure; WC, waist circumference; BMI, body mass index; FBG, fasting blood glucose; ALT, alanine aminotransferase; AST, aspartate aminotransferase; IBIL, indirect bilirubin; DBIL, direct bilirubin; ALB, albumin; GLB, globulin; UA, uric acid; CREA, creatinine; BUN, blood urea nitrogen; TC, total cholesterol; TG, triglyceride; HDL-C, high-density lipoprotein cholesterol; LDL-C, low-density lipoprotein cholesterol; HGB, hemoglobin; PLT, platelets; WBC, white blood cell; ART, antiretroviral therapy.
